# Generating evidence to narrow the treatment gap for mental disorders in sub-Saharan Africa: rationale, overview and methods of AFFIRM

**DOI:** 10.1017/S2045796015000281

**Published:** 2015-04-02

**Authors:** C. Lund, A. Alem, M. Schneider, C. Hanlon, J. Ahrens, C. Bandawe, J. Bass, A. Bhana, J. Burns, D. Chibanda, F. Cowan, T. Davies, M. Dewey, A. Fekadu, M. Freeman, S. Honikman, J. Joska, A. Kagee, R. Mayston, G. Medhin, S. Musisi, L. Myer, T. Ntulo, M. Nyatsanza, A. Ofori-Atta, I. Petersen, S. Phakathi, M. Prince, T. Shibre, D. J. Stein, L. Swartz, G. Thornicroft, M. Tomlinson, L. Wissow, E. Susser

**Affiliations:** 1 Department of Psychiatry and Mental Health, Alan J Flisher Centre for Public Mental Health, University of Cape Town, 46 Sawkins Road, Rondebosch, Cape Town, South Africa; 2 Department of Psychiatry, School of Medicine, College of Health Sciences, Addis Ababa University, Addis Ababa, Ethiopia; 3 Health Services and Population Research Department, Centre for Global Mental Health, Institute of Psychiatry, King's College London, London, UK; 4 Department of Mental Health, College of Health Sciences, University of Malawi, Blantyre, Malawi; 5 Department of Mental Health, Johns Hopkins Bloomberg School of Public Health, Baltimore, USA; 6 School of Applied Human Sciences, University of KwaZulu-Natal, Durban, South Africa; 7 Department of Psychiatry, University of KwaZulu-Natal, Durban, South Africa; 8 Department of Psychiatry, College of Health Sciences, University of Zimbabwe, Harare, Zimbabwe; 9 Centre for Sexual Health and HIV Research, University College London, London, UK; 10 Department of Health of the Government of South Africa, Pretoria, South Africa; 11 Department of Psychiatry and Mental Health, Perinatal Mental Health Project, Alan J Flisher Centre for Public Mental Health, University of Cape Town, Cape Town, South Africa; 12 Department of Psychiatry and Mental Health, University of Cape Town, Cape Town, South Africa; 13 Department of Psychology, Alan J Flisher Centre for Public Mental Health, Stellenbosch University, Stellenbosch, South Africa; 14 Aklilu-Lemma Institute of Pathobiology, Addis Ababa University, Addis Ababa, Ethiopia; 15 Department of Psychiatry, Faculty of Medicine, Makerere University, Kampala, Uganda; 16 School of Public Health and Family Medicine, University of Cape Town, Cape Town, South Africa; 17 BasicNeeds, Kampala, Uganda; 18 Department of Psychology, Faculty of Medicine, University of Ghana, Accra, Ghana; 19 Ontario Shores Center for Mental health Sciences, Ontario, Canada; 20 MRC Unit on Anxiety and Stress Disorders, Medical Research Council, Cape Town, South Africa; 21 Department of Health, Behavior, and Society, Johns Hopkins School of Public Health, Baltimore, MD, USA; 22 Mailman School of Public Health, Columbia University, New York, USA; 23 New York State Psychiatric Institute, New York, USA

**Keywords:** Mental health, multicultural, primary care, randomised controlled trials

## Abstract

There is limited evidence on the acceptability, feasibility and cost-effectiveness of task-sharing interventions to narrow the treatment gap for mental disorders in sub-Saharan Africa. The purpose of this article is to describe the rationale, aims and methods of the Africa Focus on Intervention Research for Mental health (AFFIRM) collaborative research hub. AFFIRM is investigating strategies for narrowing the treatment gap for mental disorders in sub-Saharan Africa in four areas. First, it is assessing the feasibility, acceptability and cost-effectiveness of task-sharing interventions by conducting randomised controlled trials in Ethiopia and South Africa. The AFFIRM Task-sharing for the Care of Severe mental disorders (TaSCS) trial in Ethiopia aims to determine the acceptability, affordability, effectiveness and sustainability of mental health care for people with severe mental disorder delivered by trained and supervised non-specialist, primary health care workers compared with an existing psychiatric nurse-led service. The AFFIRM trial in South Africa aims to determine the cost-effectiveness of a task-sharing counselling intervention for maternal depression, delivered by non-specialist community health workers, and to examine factors influencing the implementation of the intervention and future scale up. Second, AFFIRM is building individual and institutional capacity for intervention research in sub-Saharan Africa by providing fellowship and mentorship programmes for candidates in Ethiopia, Ghana, Malawi, Uganda and Zimbabwe. Each year five Fellowships are awarded (one to each country) to attend the MPhil in Public Mental Health, a joint postgraduate programme at the University of Cape Town and Stellenbosch University. AFFIRM also offers short courses in intervention research, and supports PhD students attached to the trials in Ethiopia and South Africa. Third, AFFIRM is collaborating with other regional National Institute of Mental Health funded hubs in Latin America, sub-Saharan Africa and south Asia, by designing and executing shared research projects related to task-sharing and narrowing the treatment gap. Finally, it is establishing a network of collaboration between researchers, non-governmental organisations and government agencies that facilitates the translation of research knowledge into policy and practice. This article describes the developmental process of this multi-site approach, and provides a narrative of challenges and opportunities that have arisen during the early phases. Crucial to the long-term sustainability of this work is the nurturing and sustaining of partnerships between African mental health researchers, policy makers, practitioners and international collaborators.

## Background

In sub-Saharan Africa, mental and substance use disorders accounted for 19% of the disability-associated disease burden (years lived with a disability, YLD) in 2010, and their relative importance is projected to increase with demographic and epidemiological transitions (Institute of Health Metrics and Evaluation, 2013; Whiteford *et al.* 2013). Mental and substance use disorders also display a high level of comorbidity with HIV, developmental disorders, epilepsy and the growing burden of non-communicable diseases (NCDs) (Prince *et al.* 2007). Despite the enormous need for mental health care, only 42% of sub-Saharan African countries have an officially adopted mental health policy, and a median of 0.62% of the health budget is spent on mental health in these countries (World Health Organization, 2011). Psychiatric hospitals remain the dominant mental health resource, with 77% of African countries’ mental health budgets spent on these facilities; the delivery of mental health services through primary care is either absent or fragmented (World Health Organization, 2011). The gap between the number of people with mental disorders who require, and those who receive treatment – the ‘treatment gap’ – is large. Current estimates range from 75% in South Africa (Williams *et al.* 2008) to over 90% in Ethiopia (Alem *et al.* 2009). It is unlikely that this treatment gap will be met by mental health specialists alone as there is approximately one psychiatrist per 2.5 million people, one psychiatric nurse per 500 000 people and one psychologist per 2 million people in Africa (World Health Organization, 2011).

There is growing international consensus that a task-sharing approach is required to narrow the treatment gap in low and middle-income countries (LMIC) (World Health Organization, 2008). According to this approach, circumscribed packages of mental health interventions are delivered by general health workers who are trained and supervised by mental health specialists, through routine health care delivery systems (Lancet Global Mental Health Group, 2007; Kakuma *et al.* 2011). Such an approach carries a number of potential advantages, including improving access to care, reduced stigma and opportunities for integrating physical and mental health care. A major challenge is that there are limited research data on the feasibility, acceptability and cost-effectiveness of such interventions in sub-Saharan Africa (Saxena *et al.* 2007). The paucity of data is partly reflective of limited capacity in African countries to design and execute research that addresses these questions (Sharan *et al.* 2007).

There is a vital need to build the evidence base for task-sharing interventions for mental health, while simultaneously developing research capacity and strengthening collaboration amongst researchers, government agencies and non-governmental organisations (NGOs). The AFrica Focus on Intervention Research for Mental health (AFFIRM) collaborative research hub was established to address these issues. As a trans-national collaboration, AFFIRM provides the kind of research approach that is fitting for the complex challenges facing mental health care in sub-Saharan Africa.

The purpose of this article is to describe the rationale, aims and methods of AFFIRM, to describe the developmental process of this multi-site approach to conducting randomised controlled trials (RCTs) of task-sharing interventions, and to provide a narrative of challenges and opportunities that have arisen during early phases. The AFFIRM consortium is one of five Collaborative Research Hubs in Global Mental Health, supported by the National Institute of Mental Health (NIMH). AFFIRM supplements the work of other current international collaborations working to expand the evidence base for scaling up mental health care in LMIC, such as the PRogramme for Improving Mental health care (PRIME) (Lund *et al.* 2012), and the Emerging Mental health systems in low and middle-income countries (EMERALD) initiative. While other current collaborations provide a focus on implementation science, in the case of PRIME, and health systems, in the case of EMERALD, the AFFIRM Hub offers a unique opportunity to generate new knowledge on the cost-effectiveness of task-sharing approaches to maternal mental health and severe mental illness that can be taken up by policy makers on the continent.

## Goal, aims and objectives of AFFIRM

**Fig. 1.**
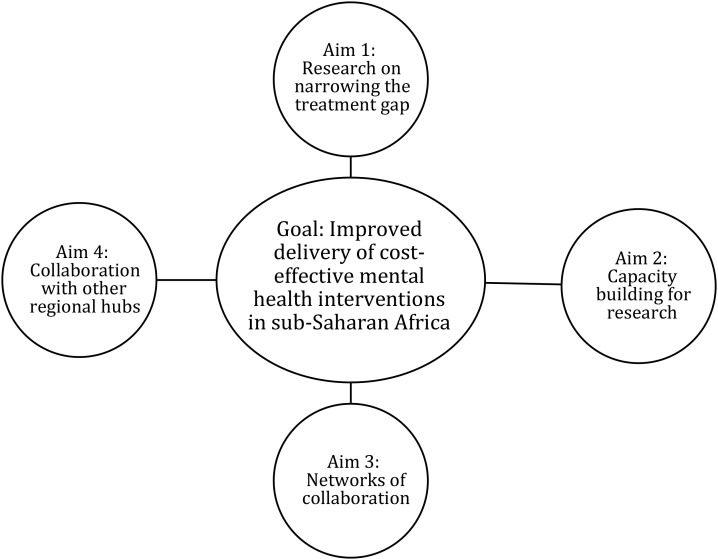
Goal and aims of AFFIRM.

The overall goal of AFFIRM is to improve the delivery of cost-effective mental health interventions in sub-Saharan Africa. AFFIRM has four main aims, each of which is served by specific objectives (see Fig. 1). The first aim is to investigate strategies for narrowing the treatment gap for mental disorders in sub-Saharan Africa by assessing the feasibility and acceptability of task-sharing interventions in Ethiopia and South Africa; and conducting randomised controlled trials (RCTs) of low cost task-sharing interventions for severe mental disorders (SMD) in Ethiopia and maternal depression in South Africa. The second aim is to build individual and institutional capacity for intervention research in sub-Saharan Africa by providing fellowship and mentorship programmes for candidates in Ethiopia, Ghana, Malawi, Uganda and Zimbabwe; offering short courses in mental health intervention research in sub-Saharan Africa; and providing supervision to Masters and PhD students who base their research in either of the AFFIRM research sites in Ethiopia and South Africa. The third aim is to collaborate with other regional NIMH hubs by designing and executing shared research projects related to task-sharing and narrowing the treatment gap; and pooling research knowledge from all hubs to contribute to global advocacy initiatives to narrow the treatment gap for mental disorders in LMIC. The fourth aim is to establish a network of collaboration between researchers, NGOs and government agencies that facilitates the translation of research knowledge into policy and practice by establishing knowledge exchange fora with Ministries of Health in all partner countries; and by producing communication materials, such as policy briefs and annual newsletters on intervention research for mental health in sub-Saharan Africa, to be disseminated to partner Ministries of Health and NGOs.

## Research component

In *Ethiopia*, improving access to care for people with SMD through task-sharing mental health care with primary care is a high priority of the National Mental Health Strategy (Federal Democratic Republic of Ethiopia Ministry of Health, 2012). Rigorous evaluation of proposed task-sharing models can provide vital evidence on whether Ethiopia's planned scale-up of mental health care is workable, acceptable, effective and cost-effective. Due to the pioneering epidemiological studies conducted in the Butajira area of south-central Ethiopia, a clinically defined, representative, rural, population-based sample of people with SMD is available for evaluation of the new task-sharing model of care. Between 1998 and 2001, community-based ascertainment of people with SMD took place using a door-to-door survey of 68 378 individuals coupled with key informant case identification (Alem *et al.* 2009; Shibre *et al.* 2014). The survey yielded a regionally unique sample of 919 people diagnosed with SMD (DSM-IV diagnoses of schizophrenia or schizoaffective disorder, bipolar disorder or major depressive disorder), of whom approximately 600 remain under follow-up after 12–15 years.

The AFFIRM Task-sharing for the Care of Severe mental disorders (TaSCS) trial in Ethiopia aims to determine the acceptability, affordability, effectiveness and sustainability of mental health care for people with SMD delivered by trained and supervised non-specialist, primary health care (PHC) workers compared with an existing psychiatric nurse-led service. The primary hypothesis is that people with SMD who receive mental health care task-shared with PHC will have a non-inferior clinical outcome, defined as a difference in the mean symptom score on the Brief Psychiatric Rating Scale, expanded version (BPRS-E) (Overall & Gorham, 1962), of no more than six points higher, compared to people with SMD who receive a psychiatric nurse-led model of mental health care, after 12 months of care under the comparison service models. The service models will also be compared on the basis of the secondary outcomes of functional impairment, relapse, cost, medication side effects and adherence, engagement in care, physical health care and nutritional status, service satisfaction, therapeutic alliance with the health care provider, quality of care, stigma, experience of restraint and adverse events such as suicide attempts.

In *South Africa*, the objective of the RCT is twofold (Lund *et al.* 2014). The first objective is to determine the effectiveness and cost-effectiveness of a task-sharing counselling intervention for maternal depression, delivered by non-specialist community health workers (CHWs). The second objective is to examine factors influencing the implementation of the task-sharing intervention and future scale up, by assessing feasibility, sustainability, quality, and safety, and by qualitative exploration of the experience of task-sharing from the perspectives of both CHWs and depressed mothers. The target population is depressed pregnant women attending antenatal clinics in Khayelitsha, a low-income township area of Cape Town. Khayelitsha has been the site of previous epidemiological studies which have reported that 39% of antenatal women scored 14 or above on the Edinburgh Postnatal Depression Scale (EPDS) (Hartley *et al.* 2011) and 34.7% of postnatal women met the criteria for DSM-IV diagnosed major depression (Cooper *et al.* 1999).

The AFFIRM RCT in South Africa is recruiting 420 depressed pregnant women from two antenatal clinics, with 210 women randomly allocated to each of the intervention and control group arms. The intervention group receives a series of six structured and manualised counselling sessions over a period of 3–4 months. CHW counsellors who deliver the intervention are trained, supervised and supported by a mental health counsellor (Clinical Social Worker). The control group receives enhanced usual care in the form of three monthly phone calls from a CHW who assesses the participant's mental health status, and provides information on available services. Referrals are made to Department of Health psychiatric services at any point in the process if the participants in either arm show indications of being severely depressed or suicidal. The primary outcome is the 17-item Hamilton Depression rating scale, and follow-up assessments are being conducted at 1 month antenatally and again at 3 and 12 months postnatally.

In both trials extensive qualitative formative and process research is being conducted to develop the interventions and assess their acceptability and feasibility; and to develop or validate instruments for use as outcome measures in the trials. The main features of the RCTs are summarised in Table 1.

**Table 1.** Main features of RCTs in Ethiopia and South Africa

	Ethiopia	South Africa
Design	Individual randomised controlled non-inferiority trial	Individual RCT
Participants	Community-ascertained sample of persons with Severe Mental Disorders (DSM IV Schizophrenia, Bipolar Disorder and Major Depressive Disorder) (*N* = 324)	Women 18 years or older attending their first antenatal clinic visit and screened for depression using the EPDS (*N* = 420)
Intervention	Task-sharing of care of stabilised people with SMD to PHC	Structured manualised face-to-face counselling delivered over 6 sessions by CHWs under the supervision of a mental health specialist (clinical social worker)
Training	10 days of training in mhGAP-IG packages (5 days of base course + 5 days of on the job training), delivered by project psychiatric nurse supported by project psychiatrist	5 days of counselling training with ongoing weekly group supervision and monthly individual supervision from the mental health specialist
Control	Psychiatric nurse-led out-patient service in Butajira hospital	3 monthly phone calls from a CHW who assesses the participant's mental health status, and provides information on available services
Fidelity measures	Post-training evaluations, observational measures of delivery of task shifted care and clinical records	
Primary effectiveness outcome measure	Symptom severity indicated by score on Brief Psychiatric Rating Scale – Expanded version (BPRS-E) at 12 months	Symptom severity indicated by score on an adapted 17-item Hamilton Depression Rating Scale at 3 months postnatal
Secondary outcome measures	• Functional impairment	• Depression symptoms
	• Relapse	• Suicidality
	• Service receipt	• Functional impairment
	• Service satisfaction	• Food insecurity
	• Body mass index	• Service receipt
	• Side effects	• Social support
	• Medication adherence	• HIV status
	• Clinic attendance	• Alcohol use
	• Stigma	• Physical and emotional abuse
	• Restraint	• Obstetric and child outcome measures at 3 and 12 months postnatal follow-up assessments only.
		• Adverse events
		• Adverse events
Timing of outcome assessments	Baseline, 12 and 18 months	Baseline, 1 month antenatal, 3 months postnatal, 12 months postnatal
Statistical analysis	Linear regression co-varying for baseline severity. Survival analysis for relapse.	Linear regression co-varying for baseline severity
Economic analysis	(1) Costs associated with delivering task-sharing, as well as the consequential impact on service use, will be analysed using multiple regression analysis (Bootstrap methods will be used if the residuals of the regression model are non-normally distributed). (2) Further regression models will include the main clinical measures so that the relationship between costs and outcomes can be assessed. (3) Incremental cost effectiveness ratios, e.g., cost per Disability Adjusted Life Year saved, will be conducted.	

## Capacity building component

Mental health research capacity and infrastructure in Africa are extremely limited, with little dedicated funding, a scarcity of trained mental health research personnel, a dearth of infrastructural support, and few opportunities for training, supervision and career development in mental health research (Thornicroft *et al.* 2012). There is thus a lack of capacity to conduct high quality intervention research, particularly RCTs, including economic evaluations and intervention development. AFFIRM aims to address these gaps by developing capacity in Africa for mental health intervention research. The Alan J Flisher Centre for Public Mental Health in the Department of Psychiatry and Mental Health, University of Cape Town and the Department of Psychology, Stellenbosch University, together with the Department of Psychiatry at Addis Ababa University co-ordinate and oversee the capacity development work of AFFIRM.

Each year five Fellowships are awarded (one each to Ethiopia, Ghana, Malawi, Uganda and Zimbabwe) to attend the MPhil in Public Mental Health, a joint postgraduate programme at the University of Cape Town and Stellenbosch University. The Fellows attend 3 weeks of intensive research methodology training after which they return to their home countries to conduct their dissertation research with the support of an academic supervisor in Cape Town or Stellenbosch and a local country academic supervisor. This approach is intended to support Fellows while they learn and work in their own countries. Regular monthly webinars are conducted on key topics to further support the Fellows during the course of the year. We envisage that the Fellowships will yield a total of 25 Masters graduates over the 5 years of the AFFIRM collaboration, creating a network of new capacity for mental health research across the partner countries. The Fellowships have also built institutional capacity by providing seed funding for the establishment of the Masters programme in Public Mental Health, which is likely to be supported in a sustained way by the University of Cape Town in future, and will provide a platform for strengthening postgraduate programmes in all AFFIRM partner institutions.

AFFIRM also offers short courses in intervention research, the latest of which was a 5-day short course on RCTs for mental health in Africa, delivered in Cape Town in November 2013 by our partners from the Centre for Global Mental Health at the Institute of Psychiatry, King's College London and Addis Ababa University.

In addition, the RCTs in Ethiopia and South Africa provide a basis for PhD studies. Currently there is one PhD student attached to each trial, examining various aspects of the intervention and instrument development work of the trials.

## Shared project component

The shared project is being conducted in collaboration with the other four regional collaborative hubs for mental health research in Latin America, sub-Saharan Africa and south Asia. The objective of the shared research project is to identify barriers and facilitators necessary for task-sharing of mental health services in LMIC. The project will use a mixed methods approach to complement the research being conducted in the hubs’ trials.

There are three specific objectives to the shared research project. The first specific objective is to use literature reviews, existing data from the formative research, pilot studies of the main trials and new qualitative data to identify perceptions of barriers and facilitators to task-sharing from multiple perspectives including service users and their families and service providers. The second specific objective is to develop and evaluate the utility of measurement tools to assess task-sharing based on the commonly identified barriers and facilitators across the hub study sites. The third specific objective is to describe the barriers and facilitators found to be relevant to each study site through case study descriptions of the qualitative and tool-testing processes. The shared research project aims to identify common lessons from diverse low resource settings and inform wider scale up of task-sharing interventions of sufficient quality to substantially improve the lives of people living with mental disorders in low resource settings.

## Networking and policy impact

The AFFIRM project has also begun to have some reach and impact on various policy processes in partner countries, together with other initiatives that are working closely with Ministries of Health, such as PRIME and EMERALD. The first Ethiopian National Mental Health Strategic Plan was adopted by the Ministry of Health in 2012 (Federal Democratic Republic of Ethiopia Ministry of Health, 2012). AFFIRM partners have been active in drafting the strategy, providing data and now pilot testing this plan, through the TaSCS trial. The South African National Mental Health Policy and Action Plan was adopted by the national Department of Health in July 2013 (Department of Health, 2013; Stein, 2014). AFFIRM partners have been active in drafting and supporting the adoption of this plan, in close collaboration with the Department of Health. The results of the AFFIRM trial in South Africa will be fed back into the further development and implementation of this action plan. AFFIRM partners have also been actively involved in the development of the draft WHO AFRO Regional Mental Health Action Plan, together with other colleagues in the region. The AFRO Mental Health Action Plan is closely aligned with the WHO Global Mental Health Action Plan, adopted by the World Health Assembly in May 2013, and provides specific targets for African countries to invest in and scale up mental health services. All of the policy engagement work of AFFIRM relies on establishing and sustaining close collaborative working relationships with key policy makers in Ministries of Health in partner countries, many of which were initiated before the onset of AFFIRM, through long standing collaborations.

## Challenges

There are several challenges implicit in the work of AFFIRM. Firstly, there are substantial logistical and ethical challenges involved in conducting behavioural trials in low resource settings. These include explaining concepts of randomisation to participants with low levels of education, the capacity of participants with SMD to give informed consent to participation, the risk of receiving inferior care through a task-sharing model, risks of suicide and other adverse events, risks of unmasking of assessment teams who live and work in the same communities as study participants, transport and other logistical challenges which hamper adherence and follow-up, the question of what is a valid compactor or control condition, adaptation and translation of measures across cultures and languages, the capacity of regulatory bodies to oversee mental health services trials, and risks of loss of confidentiality. All of these ethical risks are carefully considered in our trial protocols, and specific strategies have been adopted to mitigate the risks, with the oversight of a Data Safety and Monitoring Board, established by the NIMH for the trials. Second, there are challenges regarding support for the AFFIRM Fellows while conducting their dissertation research at a distance in their home countries – including isolation, poor internet connectivity, other work demands and local logistical difficulties. Many Fellows are working in settings that do not have a very active research tradition, and require support to build a sustainable strong research culture. Third, there are challenges with maintaining ongoing communication between AFFIRM partners due to the large geographical distances between partners and ongoing telecommunications difficulties. Finally, despite the efforts of many stakeholders, mental health remains a low priority on the policy agendas of African governments (Tomlinson & Lund, 2012), and increasing sustained advocacy and research work is required to give adequate attention to mental health.

## Conclusion

AFFIRM is a novel mental health programme in sub-Saharan Africa that integrates elements of research, capacity building, international collaboration and policy engagement. Such an approach is necessary to ensure that common problems across the continent are addressed collaboratively, while specific local needs and solutions are identified through rigorous evaluation designs, such as RCTs. AFFIRM is working to create a collaborative effort that will move the model into the field as rapidly as possible and provide a framework that can be replicated with other promising mental health approaches. The challenge remains one of stimulating interest from a range of other stakeholders who can create and evaluate task-sharing interventions in their own settings. This is the start of a long process which will need to go beyond the life of AFFIRM and will require sustained commitment from researchers, funders, policy makers, practitioners and NGOs. Next steps will involve establishing clinical trials units with expertise in mental health services research with knock-on benefits for NCDs and other health conditions.

At the conclusion of the 5-year programme in September 2016, we aim to have generated new knowledge regarding the cost-effectiveness of task-sharing interventions for SMD in Ethiopia and maternal depression in South Africa; strengthened research capacity networks across our partner countries; and together with other collaborative hubs, generated lessons regarding the barriers and facilitators for task-sharing mental health care in LMIC. A key objective is to generate policy-relevant information for the implementation and scaling up of evidence-based mental health care in sub-Saharan African countries. Crucial is the nurturing and sustaining of partnerships between African mental health researchers, policy makers, practitioners and international collaborators.
